# Current Understanding of the Neural Circuitry in the Comorbidity of Chronic Pain and Anxiety

**DOI:** 10.1155/2022/4217593

**Published:** 2022-02-15

**Authors:** Teng Chen, Jing Wang, Yan-Qing Wang, Yu-Xia Chu

**Affiliations:** ^1^Department of Integrative Medicine and Neurobiology, School of Basic Medical Sciences, Shanghai Medical College, Institute of Acupuncture Research, Institutes of Integrative Medicine, Fudan University, Shanghai, China; ^2^Department of Nephropathy, The Third Affiliated Hospital of Shenzhen University, Luohu Hospital Group, Shenzhen, China; ^3^State Key Laboratory of Medical Neurobiology and MOE Frontiers Center for Brain Science, Institutes of Brain Science, Fudan University, Shanghai, China; ^4^Shanghai Key Laboratory of Acupuncture Mechanism and Acupoint Function, Department of Aeronautics and Astronautics, Fudan University, Shanghai, China; ^5^Shanghai Research Center for Acupuncture and Meridian, Shanghai, China

## Abstract

Chronic pain patients often develop mental disorders, and anxiety disorders are common. We hypothesize that the comorbid anxiety results from an imbalance between the reward and antireward system due to persistent pain, which leads to the dysfunction of the pain and anxiety regulatory system. In this review, we will focus on changes in neuroplasticity, especially in neural circuits, during chronic pain and anxiety as observed in animal studies. Several neural circuits within specific regions of the brain, including the nucleus accumbens, lateral habenular, parabrachial nucleus, medial septum, anterior cingulate cortex, amygdala, hippocampus, medial prefrontal cortex, and bed nucleus of the stria terminalis, will be discussed based on novel findings after chemogenetic or optogenetic manipulation. We believe that these animal studies provide novel insights into human conditions and can guide clinical practice.

## 1. Introduction

Pain is an unpleasant experience that comprises sensory, emotional, and cognitive dimensions [1]. Physiological pain protects people from tissue damage, while pathologic pain such as chronic pain can lead to unnecessary suffering. Many chronic pain patients suffer from comorbid mental disorders, thus making their treatment particularly difficult, and anxiety symptoms are among the most common comorbidities in chronic pain patients [2]. Anxiety is a temporally diffused emotional state caused by a potential threat, but a threat with low likelihood of occurrence and low likelihood of producing serious harm [3]. Like pain, physiological anxiety protects people from potential danger, while pathologic anxiety leads people to overestimate the potential danger, which can impair their mental health. The lack of effective treatments is not only due to the complexity of the comorbidity, but also to a lack of understanding of the underlying mechanisms. Cumulative neuroimaging studies have shown that several different brain areas are involved in both pain and anxiety [4], but imaging studies cannot identify the causal roles of the specific regions. However, improved methods in animal models have provided an ever-greater understanding of the relationship between pain and anxiety at both the molecular, synaptic, and neural network levels. In this review, we will mainly discuss changes in neuroplasticity at the level of neurocircuits in cases of chronic pain and anxiety (Table 1).

## 2. The Hypothesis of the Comorbidity of Chronic Pain and Anxiety

The traditional hypothesis of the cooccurrence of pain and anxiety is that chronic pain causes anxiety, and that anxiety in turn exacerbates pain [2]. Zhuo has proposed that the amygdala and its related network play a key role in physiological anxiety, while the anterior cingulate cortex (ACC) and its related network are involved in pathological anxiety triggered by chronic pain. It is further argued that presynaptic long-term potentiation (LTP) in the ACC in turn plays an important role in chronic pain-induced anxiety [4]. Borsook et al. proposed a model for chronic pain called the Combined Reward Deficiency Antireward Model [5]. In this model, acute pain activates the reward system for pain relief, but failure to relieve the pain inhibits the brain's reward and motivational centers and diminishes the motivational/incentive salience of natural reinforcers, and this is referred to as a reward-deficiency state. In response to this state, the antireward system releases massive stress-related chemicals leading to diminished dopaminergic neurotransmission (reduced dopamine receptors, diminished dopamine synthesis, and increased dopamine transporters), and this is referred to as an antireward state. This maladaptive state in chronic pain enhances pain perception and comorbid changes, including addiction, depression, and anxiety.

**Figure 1:**
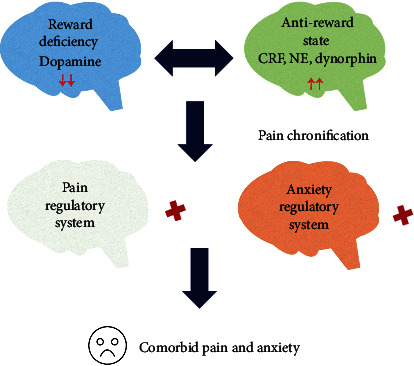
Possible explanation of the comorbidity of chronic pain and anxiety. Persistent pain inhibits the brain's reward and motivational center—the nucleus accumbens (NAc)—and diminishes the motivational/incentive salience of natural reinforcers (reward deficiency). In response to this state, the antireward system center—the lateral habenular (LHb)—is overexcited, releasing stress-related chemicals—including corticotropin-releasing factor (CRF), norepinephrine (NE), and dynorphin—leading to excessive dopaminergic trafficking (reduced dopamine receptors, diminished dopamine synthesis, and increased dopamine transporters) that results in the dysfunction of the pain and anxiety regulatory system (pain chronification). The pain and anxiety syndromes in chronic pain patients are thus the result of the dysfunction of the regulatory system.

Based on this model, we hypothesize that the comorbid anxiety originates from an imbalance in the interaction between the reward and antireward systems due to persistent pain that leads to dysfunction of the pain and anxiety regulatory system (Figure 1). Apart from stress-related chemicals—including corticotropin-releasing factor and norepinephrine—pain-induced changes in the endogenous opioid system also play an important role in mediating chronic anxiety. Kappa opioid receptor and its endogenous dynorphin are one of the key molecular elements mediating aversion [6]. Chronic pain induces kappa opioid receptor activation, which in turn inhibits dopamine release and finally results in a state of anxiety. Furthermore, it is found that kappa opioid receptor activation in the central amygdala generates both pain-like behavior [7] and anxiety-like behavior [8]. Secondly, Mu opioid receptor activity after nerve injury enhances mechanical pain sensitivity and increases anxiety-like responses, while delta opioid receptors decrease nociceptive and depressive-like behaviors after nerve injury [9], suggesting that different types of opioid systems mediate different aspects of pain and its comorbid actions. Finally, functional switching of delta opioid receptor 1 (DOR1) to delta opioid receptor 2 (DOR2) is associated with anxious states during pain chronification. DOR1 inhibits both the anxiolytic circuit from the basolateral amygdala (BLA) to the central nucleus amygdala (CeA) and the anxiogenic circuit from the parabrachial nucleus (PB) to the CeA. In contrast, activation of DOR2 mainly inhibits the PB-CeA circuit [10].

In the following sections, we first discuss the precise mechanisms of the reward deficiency and the antireward imbalance by focusing on the centers of the reward and antireward systems—the nucleus accumbens and lateral habenular, respectively. In the second part, we focus on recent studies on the dysfunction of the pain and anxiety regulatory system, including several neural circuits in the medial septum, anterior cingulate cortex, amygdala, medial prefrontal cortex, hippocampus, and the bed nucleus of the stria terminalis. Finally, we discuss future directions for studying the comorbidity between chronic pain and anxiety.

## 3. Reward Deficiency and Antireward Imbalance

### 3.1. The Nucleus Accumbens (NAc)

The NAc is composed of core and shell subregions and is a key regulator of the brain reward system, and thus, the NAc plays an important role in the regulation of chronic pain and anxiety. There are two fundamental pathways of NAc outputs—the direct pathway to striatonigral neurons marked by dopamine D1 receptors in the basal ganglia and the indirect pathway to striatopallidal neurons marked by D2 receptors in the basal ganglia. The direct pathway is considered to be correlated with reward and positive effect, while the indirect pathway is correlated with aversive events [11, 12]. It has been found that spared nerve injury (SNI) increases the excitability of NAc shell neurons that are involved in the indirect pathway, and chemogenetically inhibiting the NAc shell neurons alleviates pain-like behaviors [13]. It should be noted that this phenomenon is observed at early time points (5 days after the SNI surgery), which means that the NAc might be involved in the transition from acute pain to chronic pain but might not be responsible for the persistent state of chronic pain. It has also been reported that chronic pain elicits long-term depression of the D2 receptors expressing medium spiny neurons in the NAc core, and this phenomenon appears to be responsible for the decreased motivation that is associated with chronic pain [14]. Whole-cell recording shows that during chronic pain, the excitatory postsynaptic currents are significantly decreased in neurons expressing the D2 receptor, and the presynaptic expression of vesicular glutamate transporter1 is also reduced in the NAc [15], thus indicating that reduced glutamate release from presynaptic terminals of the NAc might be critical in the maintenance of chronic pain. A recent study found that the gene expression of *Fos*-family transcription factors in the NAc is significantly reduced after SNI of the sciatic nerve model, which is a widely used neuropathic pain model [16]. Interestingly, c-Fos transcript levels decrease only at the 5-day time point in the ipsilateral and contralateral NAc after SNI surgery, while *Δ*FosB, the stable isoform, decreases only at the 28-day time point in the contralateral NAc after SNI surgery. Moreover, unilateral overexpression of *Δ*FosB in the NAc improves neuropathic pain, indicating that *Δ*FosB serves as the long-term regulator of gene expression in persistent pain. Inhibition of the NAc during chronic pain might be explained by the suppressed ventral tegmental area (VTA), a key dopaminergic center in the brain, and it has been shown that optogenetic activation of the dopaminergic neurons in the VTA that project into the NAc core produces an analgesic effect in chronic pain [17]. Recently, it has been found that optogenetic activation of GABAergic VTA neurons induce anxiety-like behaviors [18]. Dopaminergic projections from the VTA innervate the interpeduncular nucleus (IPN), and photoinhibition or photoactivation VTA-IPN results in anxiety-like behavior or anxiolytic behavior, respectively [19].

Another important circuit within the NAc is the prelimbic area of the prefrontal cortex- (PL-) NAc core loop, and it has been reported that activating the PL-NAc circuit relieves both the sensory components and the affective syndromes of chronic pain as well as the comorbid depressive symptoms [20], while inhibition of the PL-NAc circuit amplifies sensory and affective pain [21]. It would be interesting in future work to explore the role of the PL-NAc circuit in comorbid anxiety. The infralimbic cortex- (IL-) NAc core circuitry might be responsible for pain-related anxiety, and using the pain-predicted cue- (PPC-) avoidance paradigm (PPC is the avoidance of pain using contextual cues that predict painful outcomes, and in this study, the mice were trained to associate an auditory cue with noxious stimuli, and the avoidance behavior was reflected by reduced consumption of sucrose), researchers found that chronic pain reinforced PPC avoidance while optostimulating the IL-NAc circuit suppressed PPC avoidance in a chronic pain model [22]. The increased PPC avoidance can be viewed as the result of pain-related anxiety.

### 3.2. The Lateral Habenula (LHb)

The LHb has been proposed to be the center of the antireward system. Most LHb neurons are glutamatergic with few GABAergic neurons [23]. The connectivity of the LHb is extremely complex, and the LHb receives pain signals directly from the spinal dorsal horn (SDH) or indirectly from the lateral hypothalamus (LH). The main outputs of the LHb are the dorsal raphe and median raphe, the periaqueductal gray (PAG), the VTA, and the lateral dorsal tegmental nucleus [24]. Considering that the dorsal raphe nucleus (DRN) plays a key role in descending pain modulation, a recent study found that lesion of the LHb improves the pain threshold and depression-like behaviors caused by nerve injury and increases the concentration of 5-hydroxytryptamine (5-HT) in the DRN [25]. The LHb-DRN circuit might also be important for pain-related anxiety. Another important circuit for the comorbidity of pain and anxiety might be the LHb-VTA circuit, which acts as a regulatory center for the dopaminergic system, and a study showed that either pharmacological activation or inhibition of D1 receptors in the LHb increased anxiety-like behavior while decreasing depressive-like behaviors [26]. It would be interesting to study the role of the LHb-VTA circuit in controlling pain-related anxiety using highly specific chemogenetic and optogenetic methods.

Our group recently found that bilateral inhibition of overexcited LHb glutamatergic neurons has anxiolytic and analgesic effects in the partial transection of the infraorbital nerve model, which is used to study the mechanisms of trigeminal neuropathic pain [27]. Using microarray analysis and real-time PCR, we found that gene expression in the LHb is different in chronic pain compared with the normal state, and among these, the *Tacr3* gene—which encodes the neurokinin 3 receptor that is important in the modulation of pain and negative emotion [28, 29]—is downregulated. Intriguingly, unilateral inhibition of the LHb or overexpression of the *Tacr3* gene alleviates only anxiety-like behaviors with no effect on pain-related behaviors, while bilateral overexpression of the *Tacr3* gene alleviates both. This interesting phenomenon might be explained by the left-right asymmetry of the LHb [30] because the input/output circuits between the left and right Hb have been shown to be different [31]. Considering that the LHb is divided into several functionally different subnuclei marked with various neurochemical contents [32], the failure to reduce pain unilaterally might also be correlated with different levels of inhibition of the subnuclear regions. Taken together, these results suggest that the connectivity and function of each side of the LHb and the included subnuclei should be considered in future studies.

A recent study found that 5-HT-expressing neurons in the DRN (DRN^5-HT+^) project to somatostatin- (SOM-) expressing neurons in the central amygdala (CeA^SOM+^), and the LHb is the output of the DRN^5-HT+^-CeA^SOM+^ circuit. That study found that the activity of the DRN^5-HT+^-CeA^SOM+^ circuit is decreased in the comorbid condition of pain, anxiety, and depression, and that activation of the DRN^5-HT+^-CeA^SOM+^-LHb circuit could reverse the nerve injury-induced reduction in pain threshold and the depression-like behavior [33]. Because this group focused on the comorbidity of pain and depression, they did not check the role of the DRN^5-HT+^-CeA^SOM+^-LHb circuit in anxiety-like behaviors. Considering that the CeA and LHb are crucial for anxiety, the DRN^5-HT+^-CeA^SOM+^-LHb circuit might also be important for the comorbidity of pain and anxiety.

## 4. Dysfunction of the Pain and Anxiety Regulatory System

### 4.1. The Parabrachial Nucleus (PB)

The PB is known to relay pain-related information from the spinal cord in the affective pain pathway. The PB-nociceptive neurons project to multiple emotion and instinct-related centers, including the bed nucleus of the stria terminalis (BNST), the paraventricular thalamic nucleus, the paraventricular nucleus of the hypothalamus, the CeA, the ventral tegmental area, the ventrolateral periaqueductal grey, the nucleus of the solitary tract, and the intermediate reticular nucleus in the hindbrain [34]. A recent study found that short-term optogenetic activation of GABAergic lateral PB neurons or inhibition of glutamatergic lateral PB neurons alleviates pain-like behavior in a common peroneal nerve ligation model, but short-term activation of glutamatergic lateral PB neurons is not sufficient to induce anxiety-like behaviors [35]. Also, activation of the lateral PB to the BNST or CeA generates an aversive learning to noxious stimulation, while activation of the ventromedial hypothalamus or lateral periaqueductal gray drives escape behaviors [36].

The CeA receives direct nociceptive information from the PB via the spino-ponto-amygdaloid pathway that relays the pain signals from the spinal cord to the CeA. Activating the PB-CeA pathway to stimulate pain signals in normal mice produces anxiety-like behaviors with no influence on pain responses [37]. Two subtypes of CeA neurons that are targeted by the PB and that mediate opposing effects after nerve injury have been identified by molecular genetic approaches [38]. CeA neurons expressing protein kinase C-delta (Ce^APKC*δ*+^) display hyperexcitability and promote pain-like behaviors after nerve injury, while CeA^SOM+^ display hypoexcitability and drive antinociception. Because both types of cells receive excitatory inputs from the PB, it is believed that changes in the outputs of these cells are the main cause of pain-related plasticity. The antinociceptive effect of CeA^SOM+^ neurons has recently been found to be related to the CeA^SOM+^-PB pathway, and a study showed that CeA^SOM+^ neurons send inhibitory GABAergic inputs into the PB [39]. The CeA^SOM+^-PB circuit is weakened under conditions of chronic pain, thus overexciting PB neurons and resulting in pain-like behaviors. A recent group challenged the traditional notion that the PB conveys nociceptive information directly to the CeA. Inconsistently, they found that *Tacr1^+^* neurons in the PB represent the major target of spinal projection, which projects directly to the intralaminar thalamic nuclei but not the CeA [40]. This might be related to differential projections of the dorsal lateral PB versus the external lateral PB.

### 4.2. The Medial Septum (MS)

The MS consists of cholinergic neurons and receives noxious stimuli from widespread peripheral regions and then projects to a broad range of pain-modulatory sites in the neocortex such as the ACC and the hippocampus, thus playing a vital role in various cognitive and emotional behaviors. A recent study showed that chemogenetic inhibition of MS cholinergic neurons (MS^choli+^) and the MS^choli+^-rostral ACC (rACC) circuit alleviates chronic inflammatory pain-induced anxiety-like behaviors in the elevated plus maze (EPM) and the open-field test (OFT) [41]. The EPM is conducted in a plus-shaped maze with four arms, two of which are enclosed by high walls, called the closed arms, and two of which are left open, called the open arms. Animals making fewer entries into and spending less time exploring the aversive open arms are considered to be exhibiting anxiety-like behaviors. The OFT consists of a square arena enclosed by high walls, and animals spending less time exploring the center of the arena are considered to be exhibiting anxiety-like behavior [42]. The same group also found that inhibiting the MS^choli+^-rACC or MS^choli+^-ventral CA1 (vCA1) circuit has an analgesic effect [43]. Interestingly, the authors found that chemogenetic inhibition of the MS^choli+^-vCA1 circuit seems to have no effect on pain-related anxiety [41]. One explanation for this is that persistent chronic pain can impair the activity of the hippocampus [44], thus making the MS-vCA1 circuit ineffective.

### 4.3. The ACC

The ACC is involved in both pain perception and anxiety [4]. Two forms of LTP, a form of synaptic plasticity, have been identified in the ACC—a presynaptic form (pre-LTP) that requires kainate receptors and a postsynaptic form (post-LTP) that requires N-methyl-D-aspartate receptors. Surprisingly, pharmacological inhibition of pre-LTP has anxiolytic and analgesic effects in chronic pain models [45], while pharmacological inhibition of post-LTP has only analgesic effects [46]. Therefore, it has been proposed that pre-LTP is the primary mechanism through which the ACC mediates pain-induced anxiety (for detailed mechanisms of the pre-LTP, see Zhuo's review about long-term cortical synaptic changes [47]) However, considering the diverging output and input of ACC neurons and considering the limitations of pharmacological methods, even though pre-LTP is crucial for pain-induced anxiety, some ACC neurons might not depend on pre-LTP but might still be important for pain-induced anxiety for specific neural connections.

There are several neural circuits that are important for understanding the comorbidity of pain and anxiety. First, the ACC neurons form bidirectional innervations with the amygdala, a central region in anxiety modulation, both directly (the ACC-amygdala circuit) and indirectly (the ACC-thalamus-amygdala circuit) [4]. Optical inhibition of the ACC decreases abnormal ventral posteromedial thalamus activities, which increases the pain response in a chronic constriction injury of the infraorbital nerve model [48]. Second, the ACC-PAG-rostromedial ventral medulla- (RVM-) SDH pathway, known as the descending pain regulation system, and the ACC-SDH pathway complementarily regulate nociceptive sensory transmission [49]. It is possible that the traditional pain-related circuits might convert into anxiety-related circuits under comorbid conditions or might have some as yet undiscovered anxiety-modulating effect due to limitations in experimental methods. More work thus needs to be done to explore the possible role of these circuits in pain-anxiety comorbidity.

### 4.4. The Amygdala

The amygdala, including the BLA and the CeA, is important for emotional processing and has a role in sensations of pain and feelings of anxiety [50]. The CeA encompasses the centrolateral (CeL) and centromedial (CeM) nuclei, and the CeM is the primary output region of the amygdala. A recent study showed that CeA^SOM+^ neurons in CeL mediate anxiety-like behavior by inhibiting GABAergic neurons in the central sublenticular extended amygdala [51]. Optogenetic activation of a distinct group of GABAergic neurons in the CeA, which can be activated by general anesthesia, inhibits pain-like behaviors in normal and chronic pain states [52]. The CeA received projections from the posterior thalamic paraventricular nucleus (pPVT), and stimulating the pPVT-CeA pathway induces pain-like behavior [53].

About 90% of the BLA consists of glutamatergic neurons, while about 95% of the CeA consists of GABAergic neurons. Optogenetic stimulation of BLA termini in the CeA decreases anxiety-related behaviors in both the EPM and the OFT [54], and it is hypothesized that BLA neurons excite GABAergic CeL neurons that then exert feed-forward inhibition onto CeM output neurons to produce the anxiolytic effect [54]. Because the BLA is a site for converging negative and positive stimuli and because of the functional differences in the anterior and posterior BLA (aBLA and pBLA), it is argued that the BLA neurons that contribute to positive behaviors (positive neurons) and negative behaviors (negative neurons) might be genetically distinguishable. The putative negative neurons are targeted by exposing male mice to foot shocks, and positive neurons are targeted by exposing male mice to a female mouse. Utilizing a c-Fos-based genetic expression system, *Ppp1r1b*- (protein phosphatase 1 regulatory inhibitor subunit 1B-) expressing neurons were identified as positive neurons mainly located in the pBLA, and *Rspo2*- (R-spondin 2-) expressing BLA neurons were identified as negative neurons in the aBLA [55]. Contrary to a previous hypothesis, retrograde and anterograde tracing suggests that *Ppp1r1b*^+^ BLA neurons make distinct projections to the CeL, CeM, and infralimbic cortex, while *Rspo2*^+^ neurons make distinct projections to the capsular nucleus of the central amygdala and prelimbic cortex [55]. Although *Ppp1r1b* and *Rspo2* can distinguish between positive and negative BLA neurons, it should be noted that there might be other genetic markers and that *Ppp1r1b^+^* and *Rspo2^+^* neurons can be further divided into subgroups based on specific behavior paradigms and by using improved analytic methods. Therefore, this hypothesis should be modified to suggest that *Ppp1r1b*^+^ BLA neurons, which are positive neurons, excite the CeL neurons that exert feed-forward inhibition onto CeM output neurons and finally produce the anxiolytic effect. Interestingly, activating the BLA-CeA pathway in a chronic inflammatory pain model alleviates both mechanical and thermal pain responses [37].

Recently, a distinct group of neurons that encode the negative affective valence of pain has been found in the BLA [56]. Normally, silencing this nociceptive ensemble reduces attending and escape behaviors in response to noxious stimuli without changing stimulus detection, withdrawal, anxiety, or reward. Moreover, this nociceptive ensemble that is normally activated by noxious stimuli can also be activated by innocuous stimuli during conditions of chronic pain. It thus seems reasonable to hypothesize that this ensemble contributes to pain-related anxiety as a result of dysfunctional perceptual changes. The BLA-medial prefrontal cortex- (mPFC-) PAG-SDH circuit is crucial for the development of neuropathic pain, and it has been found that increased inputs from the BLA to the mPFC GABAergic interneurons—which send feed-forward inhibition to pyramidal neurons in the mPFC—induced by nerve injury lead to the net inhibition of mPFC output [57]. Therefore, feed-forward inhibition of the downstream mPFC-PAG-SDH circuit occurs. One thing to be noted is that even though inhibiting the BLA-mPFC circuit alleviates pain responses in SNI mice, the pain-related anxiety is insensitive to the same manipulation, indicating that there might be other pathways within the BLA that mediate pain-related anxiety.

In addition to the BLA-CeL-CeM circuit, the BLA-ventral hippocampus (vHPC) circuit also plays a vital role in modulating anxiety-related behaviors [58]. A recent study showed that the aBLA and pBLA innervate the deep-layer calbindin1- (Calb1-) negative and superficial-layer Calb1-positive neurons in the vCA1, respectively, and thus, the aBLA-vCA1^Calb1−^ circuit drives avoidance behavior and exerts anxiogenic effects, while the pBLA-vCA1^Calb1+^ circuit drives approach behavior and exerts anxiolytic effects [59]. The functional diversity along the anterior–posterior axis of the BLA is based on genetic spatiality, and the genetic spatiality of the vCA1 also exerts different effects. Calb1, a Ca^2+^ binding protein, functions as a buffer, transporter, and sensor of Ca^2+^ [60], and the different Ca^2+^ signals, which are important in a wide range of cellular functions, are spatially and temporally modified depending on the Ca^2+^ binding property and the intracellular concentration of the buffer [61]. Calb1 knockdown in the vCA1 abolishes the anxiolytic effect of the pBLA-vCA1 circuit [59], and thus, both the input specificity and the Calb1 levels determine the specific circuit-associated amelioration of anxiety. It would be interesting to identify the differences between the information transmitted by vCA1^Calb1+^ neurons and vCA1^Calb1−^ neurons, and thus, what kind of information depends on Calb1, and to identify the downstream effects of the different circuits.

### 4.5. The Hippocampus

While the hippocampus is known to be a cognitive structure involved in memory, it is also implicated in controlling emotions such as anxiety. Chronic pain causes memory deficits and atrophy of CA1 pyramidal neurons, and an increase in the dendritic tree complexity of the dentate gyrus hippocampal subregions after nerve injury has been observed [62]. Neuroimaging confirms that reduced connectivity in the hippocampus is associated with the transition from acute pain to chronic pain [63], and a recent study showed that peripheral inflammation-induced spontaneous pain disrupts vCA1-IL connectivity, while optogenetic activation of the vCA1-IL relieves pain [64]. Thus, impairment of the hippocampus from chronic pain leads to the failure of the anxiety-related modulatory system within the hippocampus.

There is functional heterogeneity along the dorsoventral axis of the hippocampus, and gene expression within the dorsal hippocampus (dHPC) correlates with cognitive functions such as learning and memory, while the ventral hippocampus (vHPC) is involved in emotional regulation [65]. The septohippocampal pathway plays an important role in controlling anxiety responses, and it has long been postulated that the hippocampus monitors the environment and sends contextual information regarding conflict and novelty to the septum in order to control anxiety [66]. The septum that projects to the hippocampus contains cholinergic, GABAergic, and glutamatergic neurons, while the hippocampus projects to the septum mainly via glutamatergic afferents. An early study found that disconnection of the lateral septum (LS) and the vHPC using asymmetric pharmacological inhibition reduced anxiety-related behaviors in the EPM, indicating that the vHPC and the LS work in tandem to modulate anxiety [67], and a recent study found that chemogenetic activation of the vHPC cells that project to the LS decreased anxiety-related behaviors [68]. Although chemogenetic techniques can selectively manipulate the vHPC-LS circuit by injecting the retrogradely propagating canine adenovirus encoding Cre recombinase into the LS and injecting the Cre-responsive adeno-associated virus into the vHPC, it should be noted that retrograde-targeted vHPC cells also send axon collaterals to other structures. The authors found that the LS-projecting vHPC cells were most abundant in the LS but less so in the dorsal CA1 and the BLA. Therefore, the behavioral change relies on the combined effects of altering multiple axon collaterals. The LS is thought to regulate anxiety through its outputs to the hypothalamus, and a recent study showed that a subset of GABAergic LS neurons expressing type 2 corticotropin-releasing factor receptor (*Crfr2*) project to the anterior hypothalamic area (AHA) of the medial hypothalamus, and that optogenetic stimulation of the LS*^Crfr2+^*-AHA circuit promotes anxiety-related behaviors and increases corticosterone levels [69]. Subsequent experiments showed that the LS*^Crfr2+^* neurons form inhibitory synapses with the AHA neurons that project to the paraventricular nucleus, which is part of the hypothalamic-pituitary-adrenocortical (HPA) axis and regulates corticosterone release. Therefore, the anxiogenic role of the LS*^Crfr2+^*-AHA circuit is due to disinhibiting the HPA axis. Because the LS*^Crfr2+^* output is anxiogenic, while the vHPC-LS circuit is anxiolytic, it is possible that the vHPC might innervate a distinct population of LS neurons that in turn inhibit the LS*^Crfr2+^* neurons and ultimately downregulate the HPA axis. However, we cannot rule out the possibility that the LS might also contain an anxiolytic subpopulation that might be activated by the vHPC. A study has shown that excitotoxic ablation of LS neurons can enhance HPA axis responses [70], thus confirming the existence of anxiolytic LS neurons, but the existence of the anxiolytic output remains to be determined.

Interestingly, LS*^Crfr2+^* neurons also make connections with the midbrain PAG, a region known to regulate defensive behaviors relevant to anxiety, via their projections to the AHA [69]. Retrogradely targeted LS-projecting vHPC cells are distributed in the ventral CA3 (vCA3) subregion and the vCA1 [68], but unlike the vCA1, the vCA3 receives glutamatergic inputs from the ventral dentate gyrus (vDG), and a recent study has shown that optogenetically stimulating the vDG suppresses anxiety-related behaviors and has no effect on contextual learning [71], while the anxiolytic effect of vDG activation might be caused by the vCA3-LS circuit. Future work should determine the different roles of the vCA1-LS and vCA3-LS circuits. In addition to the indirect vHPC-LS-AHA circuit, the vCA1 also directly projects to the LH, and a study has shown that activation of the vCA1-LH circuit can increase anxiety and avoidance behaviors [72]. The vCA1-LH can be a direct way for the vHPC to rapidly control anxiety-like behaviors, but how the vHPC-LS-AHA and the vCA1-LH coordinate with each other when animals feel anxiety needs to be determined.

In addition to the LS and LH, the vHPC also projects directly to the mPFC, which is implicated in the regulation and expression of defensive behaviors in rodents. Theta frequency (4–12 Hz) activity in the mPFC and vHPC synchronizes and increases during exposure to anxiogenic arenas [73]. Single units in the mPFC that are synchronized with the vHPC are involved in the anxiety-related behavior in the EPM [74], and subsets of vCA1 neurons projecting to the mPFC change their firing patterns under conditions of elevated anxiety [75]. In addition, optogenetic inhibition of the vHPC-mPFC circuit reduces anxiety-like behavior and the spatial representations of aversion and anxiety valence in mPFC neurons [74]. Taken together, these observations suggest that the vHPC conveys valence information to the mPFC and then the mPFC regulates the anxiety-related activities that guide the animal's anxiety-related behavior in the EPM. Classically speaking, the dHPC seems to be less involved in regulating emotional control. A recent study showed that overexpressing extracellular signal-regulated kinase-2, a signaling molecule known to regulate gene expression, in the dHPC downregulates anxiety-related behaviors in the EPM [76]. Contrary to the anxiolytic role of the dHPC, another study found that stimulation of the median raphe nucleus (MRN) 5-HT-positive neurons together with 5-HT-positive neuron innervation to the dHPC promotes anxiety-like behaviors [77]. Somewhat paradoxically, although photostimulation of MRN 5-HT-positive neurons produces anxiety-like behavior in a variety of behavioral tests, photostimulation of 5-HT-positive neuron terminals in the dHPC produces anxiety-like behaviors only in the novelty-suppressed feeding and marble-burying tests and not in the EPM. First, this might be explained by different anxiety-related paradigms and specific contributions of 5-HT-positive neuron terminals in other structures. Second, these experiments indicate that different regulation of vHPC neurons is enough to decrease or increase anxiety in a persistent manner, but they do not address whether the activity of these neurons is normally required for anxiety. Last but not least, the role of the dHPC in anxiety might not be through emotional management, but perhaps it can influence anxiety-related behaviors, and further experiments such as *in vivo* Ca^2+^ imaging should be performed to confirm the role of the dHPC in anxiety. Taken together, the work described here suggests that dysfunction of the anxiety regulatory system within the hippocampus might explain the comorbidity of anxiety with chronic pain.

### 4.6. The Bed Nucleus of the Stria Terminalis (BNST)

The BNST has been implicated in pathological and adaptive anxiety [78], and there is heterogeneity among the BNST neurons that regulate negative emotional states. The anterodorsal part of the BNST (adBNST) receives projections from the aBLA, but contrary to the vCA1, the aBLA-adBNST circuit decreases anxiety-related states when photostimulated [79]. The adBNST projections to the LH, the PB, and the VTA mediate the different features of the anxiolytic effect of the aBLA-adBNST as indicated by reduced risk avoidance, reduced respiratory rate, and increased positive valence, respectively [79]. The dorsolateral BNST (dlBNST) neurons form GABAergic connections with the CeA, and stimulating the dlBNST-CeA produces the opposite effect of stimulating the adBNST-LH circuit [80]. A recent study confirmed that CeA neurons release corticotropin-releasing factor (CRF) that binds to CRF1 receptors on dlBNST neurons resulting in anxiety-like behaviors [81]. Under conditions of emotional pain, CRF excites dlBNST neurons through adenylate cyclase-cyclic AMP-protein kinase A signaling, thus resulting in pain-induced aversion [82], and this might be the same pathway for regulating anxiety. The same group found that enhanced CRF signaling within the dlBNST also suppresses the dlBNST-VTA circuit through increased inhibitory input under conditions of chronic pain [83]. The CRF-positive neurons in the BNST (BNST^CRF+^) also receive projections from DRN^5-HT+^ neurons via 5-HT_2C_ receptors, and a study showed that stimulating the DRN^5-HT+^-BNST^CRF+^ circuit results in enhanced anxiety-like behaviors by inhibiting the anxiolytic ventral BNST- (vBNST-) LH and vBNST-VTA circuits [84]. Photostimulation of the vBNST glutamatergic projections (vBNST^Glu+^) to the VTA results in aversive and anxiogenic behaviors, while stimulating the GABAergic vBNST (vBNST^GABA+^) projections to the VTA results in rewarding and anxiolytic behaviors [85]. The vBNST also plays a vital role in the affective component of pain [86]. The vBNST receives noradrenergic projections from the medullary A1/A2 cell groups, and noradrenaline acts on *α*2-adrenoceptors and *β*-adrenoceptors located in the vBNST and thereby mediates pain-induced aversion [87, 88]. It would therefore be interesting to explore the role of the vBNST^Glu^-VTA and the vBNST^GABA^-VTA circuits in emotional pain using the conditioned place aversion paradigm. Recently, a novel circuit was found that GABAergic adBNST neurons projected directly to the parvalbumin interneurons in the shell NAc, which has an inhibitory influence on anxiety-like responses when activated [89].

Long-lasting plasticity in the BNST might also promote the comorbidity of chronic pain and anxiety. Researchers found that the IL and ventral subiculum/CA1 (vSUB/CA1) neurons project to the same anteromedial BNST (amBNST) neurons. Interestingly, vSUB/CA1-amBNST synapses promote NMDA-dependent LTP with NMDA-independent long-term depression in IL-amBNST synapses when stimulated by high frequency *in vivo*, while the same protocol in the IL fails to change the plasticity of the IL or the vSUB/CA1. *In vivo* LTP in the amBNST reduces the anxiety induced by anxiogenic situations [90]. An interesting question to address is whether the high-frequency stimulation of the vSUB/CA1 is enough to trigger plasticity changes in other anxiety-related areas like the BLA or mPFC because the vSUB/CA1 is one of the major output structures of the hippocampal signals in a high-frequency bursting mode. Taken together, studies of the BNST suggest that anxiety and the affective component of chronic pain might share the same mechanisms, which means that anxiety and emotional pain might be different representations of the same neural network changes.

## 5. Concluding Remarks and Future Directions

**Figure 2:**
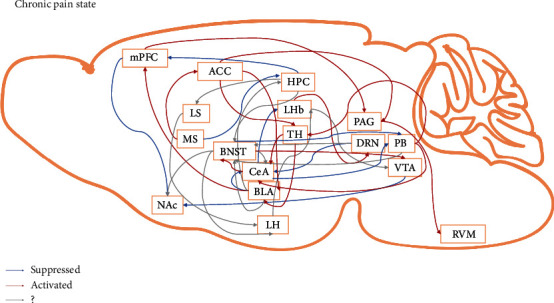
Potential neural circuits underlying the comorbidity of chronic pain and anxiety. ACC: anterior cingulate cortex; BLA: basolateral amygdala; BNST: bed nucleus of the stria terminalis; CeA: central amygdala; DRN: dorsal raphe nucleus; HPC: hippocampus; LH: lateral hypothalamus; LHb: lateral habenula; LS: lateral septum; mPFC: medial prefrontal cortex; MS: medial septum; NAc: nucleus accumbens; PAG: periaqueductal gray; PB: parabrachial nucleus; RVM: rostromedial ventral medulla; TH: thalamus; VTA: ventral tegmental area. ?, not confirmed for chronic pain-related anxiety.

In summary, the research presented here expands our knowledge of the comorbidity of chronic pain and anxiety. From our perspective, the comorbidity of chronic pain and anxiety is the result of dysfunction in the pain and anxiety regulatory systems, which means that specific dysfunctions in the functional and structural connectivity of the neural circuits that govern sensory, emotional, and cognitive functions define the unique biotypes of pain and anxiety (Figure 2). However, there are still some questions that need to be answered. First, under physiological conditions, some circuits that are normally considered to regulate anxiety seem to be involved in pain regulation. For example, studies show that activating the BLA-CeA circuit, which is generally an anxiolytic circuit, under physiological conditions alleviates thermal pain with no effect on mechanical pain [37]. The authors explain that these differential effects might reflect the different features of the two pain tests, where the thermal pain test measures paw withdrawal responses to an infrared heat stimulus (a noxious stimulus), while the mechanical pain test measures paw withdrawal responses to *von Frey* filaments (a nonnoxious stimulus). This indicates that the anxiety regulatory system and pain regulatory system partly share common circuits. In clinical practice, chronic pain patients are found to easily develop anxiety, while some anxiety disorder patients easily develop pain syndromes [2]. Therefore, the original hypothesis that pain induces anxiety and that anxiety in turn exacerbates pain might be inappropriate considering that pain and anxiety might be different representations of the same system. It would therefore be interesting to study the classical pain regulatory system in regulating anxiety behaviors. Second, some studies have focused on the mechanisms of depressive syndromes in chronic pain [20, 33], and some circuits involved in comorbid depression might also regulate comorbid anxiety. In addition, it is important to elucidate the difference between comorbid depression and comorbid anxiety because even though both syndromes can be observed in chronic pain patients, some individuals might develop comorbid anxiety, some might develop comorbid depression, and some might develop both. Thus, in future studies, it would be worthwhile to dissect the different phenotypes of chronic pain models based on behavioral tests and to study the mechanisms behind the different phenotypes. Third, the behavioral paradigm for studying comorbidity needs to be improved. Some research has demonstrated the relief of chronic pain-related anxiety using the EPM and OPT, but they are not able to explain whether or not the anxiolytic effect is based on approach-avoidance decision-making, in other words, whether the improved behavior is the result of enhanced approaching behaviors or to reduced aversive behaviors or to reduced pain perception. A novel behavioral test called the L-type elevated maze that consists of one open arm and one closed arm has been used in anxiety studies [59], and compared with the EPM, the L-type maze has a definite movement direction such that animals only have two choices, to go in the opposite direction or return to the previous direction, and this will help investigators accurately determine the animals' decision-making behaviors. We recommend this paradigm for future studies of comorbid anxiety. Fourth, more and more studies show that even in the same brain area, there is functional diversity and different connectivity. Genetic marking of these circuits is supposed to be effective in dissecting them, but specifically, marked neurons also exhibit differences. Single cell analysis might be helpful for explaining the variance and for categorizing these neurons into more distinct groups. Last but not the least, the symptoms that characterize mental disease are the result of dysfunctions within and between these circuits. However, language about brain circuits has not been incorporated into clinically meaningful taxonomies for clinical practice, and the development of a neural circuit taxonomy suited to clinical actions is needed [91]. We hope the neural circuits identified in the comorbidity of pain and anxiety from animal studies will provide novel insights into human conditions and will guide clinical practice.

## Figures and Tables

**Table 1:** Neural circuits that serve to promote or inhibit pain and anxiety. Summary of the current understanding of the neural circuits involved in or potentially involved in the comorbidity of chronic pain and anxiety. Pain-like behaviors can be measured in two aspects—sensory pain and affective pain. We measure sensory pain via withdrawal thresholds or latencies, and we measure affective pain via conditioned place avoidance or preference. ?, not confirmed; ×, no effect.

Summary				
Neural circuits	Chronic pain state	Pain (sensory or affective pain)	Anxiety	Reference
3.1 The nucleus accumbens (NAc)				
VTA-NAc	Suppressed	Analgesic when activated (sensory pain)	?	[17]
VTA-IPN	Suppressed	?	Anxiolytic when activated	[19]
PL-NAc	Suppressed	Analgesic when activated (sensory pain and affective pain)	?	[20]
IL-NAc	Suppressed	?	Anxiolytic when activated	[22]
3.2 The lateral habenula (LHb)				
LHb-DRN	Activated	Analgesic when inhibited (sensory pain)	?	[25]
LHb-VTA	?	?	Anxiogenic when activated	[26]
DRN^5-HT+^-CeA^SOM+^-LHb	Suppressed	Analgesic when activated (sensory pain)	?	[33]
4.1 Parabrachial nucleus (PB)				
Lateral PB GABA+-lateral PB Glu+	Suppressed	Analgesic when activated (sensory pain and affective pain)	×	[35]
Lateral PB-BNST	?	Generate aversive learning	?	[36]
Lateral PB-VMH	?	Drives escape behaviors	?	[36]
Lateral PB-lPAG	?	Drives escape behaviors	?	[36]
Lateral PB-CeA	Activated	No effect on sensory pain and generate aversive learning	Anxiogenic when activated	[36, 37]
CeA^SOM+^-lateral PB	Suppressed	Analgesic when activated (sensory pain)	?	[39]
Lateral PB Tacr1^+^-ILN	Activated	Promote pain when activated	?	[40]
4.2 The medial septum (MS)				
MS^choli+^-rACC	Activated	Analgesic when inhibited (sensory pain and affective pain)	Anxiolytic when inhibited	[41, 43]
MS^choli+^-vCA1	Suppressed	Analgesic when activated (sensory pain and affective pain)	×	[41]
4.3 ACC				
ACC-amygdala	Activated	Analgesic when inhibited (sensory pain and affective pain)	?	[4]
ACC-thalamus-amygdala	Activated	Analgesic when inhibited (sensory pain)	?	[48]
ACC-PAG-RVM-SDH	Activated	Analgesic when inhibited (sensory pain and affective pain)	?	[49]
4.4 The amygdala				
CeA^SOM+^-cSEA	?	?	Anxiogenic when activated	[51]
pPVT-CeA	Activated	Promote pain when activated (sensory pain)	?	[53]
BLA-CeA	Suppressed	Analgesic when activated (sensory pain)	Anxiolytic when activated	[37, 54]
BLA-mPFC-PAG-SDH	Activated	Promote pain when activated (sensory pain)	×	[57]
aBLA-vCA1^Calb1−^	?	?	Anxiogenic when activated	[59]
pBLA-vCA1^Calb1+^	?	?	Anxiolytic when activated	[59]
4.5 The hippocampus				
vCA1-IL	Suppressed	Analgesic when activated (sensory pain and affective pain)	Anxiolytic when activated	[64]
vHPC-LS	?	?	Anxiolytic when activated	[68]
LS^Crfr2+^-AHA	?	?	Anxiogenic when activated	[69]
vCA1-LH	?	?	Anxiogenic when activated	[72]
vHPC-mPFC	?	?	Anxiolytic when inhibited	[74]
MRN 5-HT+-dHPC	?	?	Anxiogenic when activated	[77]
4.6 The bed nucleus of the stria terminalis (BNST)				
aBLA-adBNST	?	?	Anxiolytic when activated	[79]
dlBNST-CeA	?	?	Anxiogenic when activated	[80]
CeA-dlBNST	Activated	Promote pain when activated (sensory pain)	Anxiogenic when activated	[81, 82]
dlBNST-VTA	Activated	?	?	[83]
DRN^5-HT+^-BNST^CRF+^	?	?	Anxiogenic when activated	[84]
vBNST^Glu+^-VTA	?	?	Anxiogenic when activated	[85]
vBNST^GABA+^-VTA	?	?	Anxiolytic when activated	[85]
Medullary A1/A2 cell groups-vBNST	Activated	Promote pain when activated (sensory pain)	?	[87, 88]
adBNST GABA+-NAc shell	?	?	Anxiolytic when activated	[89]
vSUB/CA1-amBNST	?	?	Anxiolytic when activated	[90]

NAc: nucleus accumbens; VTA: ventral tegmental area; IPN: interpeduncular nucleus; PL: prefrontal cortex; IL: infralimbic cortex; LHb: lateral habenula; DRN: dorsal raphe nucleus; CeA: central amygdala; PB: parabrachial nucleus; BNST: bed nucleus stria terminalis; VMH: ventromedial hypothalamus; lPAG: lateral PAG; ILN: intralaminar thalamic nuclei; MS: medial septum; ACC: anterior cingulate cortex; rACC: rostral ACC; PAG: periaqueductal gray; RVM: rostromedial ventral medulla; SDH: spinal dorsal horn; pPVT: posterior thalamic paraventricular nucleus; BLA: basolateral amygdala; cSEA: central sublenticular extended amygdala; mPFC: medial prefrontal cortex; vCA1: ventral CA1; vHPC: ventral hippocampus; LS: lateral septum; AHA: anterior hypothalamic area; LH: lateral hypothalamus; MRN: median raphe nucleus; dHPC: dorsal HPC; aBLA: anterior BLA; adBNST: anterodorsal part of the BNST; dlBNST: dorsolateral BNST; amBNST: anteromedial BNST; vBNST: ventral BNST; vSUB/CA1: ventral subiculum/CA1.
